# Vitamin D treatment of peripheral blood mononuclear cells modulated immune activation and reduced susceptibility to HIV-1 infection of CD4^+^ T lymphocytes

**DOI:** 10.1371/journal.pone.0222878

**Published:** 2019-09-24

**Authors:** Sandra M. Gonzalez, Wbeimar Aguilar-Jimenez, Edison Trujillo-Gil, Wildeman Zapata, Ruey-Chyi Su, T. Blake Ball, Maria T. Rugeles

**Affiliations:** 1 Grupo Inmunovirología, Facultad de Medicina, Universidad de Antioquia UdeA, Medellín, Colombia; 2 National HIV and Retrovirology Laboratory, JC Wilt Infectious Diseases Research Centre, Public Health Agency of Canada, Winnipeg, Manitoba, Canada; 3 Grupo Infettare, Facultad de Medicina, Universidad Cooperativa de Colombia, Medellín, Colombia; 4 Department of Medical Microbiology and Infectious Diseases, University of Manitoba, Winnipeg, Manitoba, Canada; University Hospital Tuebingen, GERMANY

## Abstract

**Introduction:**

Mucosal immune activation, in the context of sexual transmission of HIV-1 infection, is crucial, as the increased presence of activated T cells enhance susceptibility to infection. In this regard, it has been proposed that immunomodulatory compounds capable of modulating immune activation, such as Vitamin D (VitD) may reduce HIV-1 transmission and might be used as a safe and cost-effective strategy for prevention. Considering this, we examined the *in vitro* effect of the treatment of peripheral blood mononuclear cells (PBMCs) with the active form of VitD, calcitriol, on cellular activation, function and susceptibility of CD4^+^ T cells to HIV-1 infection.

**Methods:**

We treated PBMCs from healthy HIV unexposed individuals (Co-HC) and frequently exposed, HIV-1 seronegative individuals (HESNs) from Colombia and from healthy non-exposed individuals from Canada (Ca-HC) with calcitriol and performed *in vitro* HIV-1 infection assays using X4- and R5-tropic HIV-1 strains respectively. In addition, we evaluated the activation and function of T cells and the expression of viral co-receptors, and select antiviral genes following calcitriol treatment.

**Results:**

Calcitriol reduced the frequency of infected CD4^+^ T cells and the number of viral particles per cell, for both, X4- and R5-tropic viruses tested in the Co-HC and the Ca-HC, respectively, but not in HESNs. Furthermore, in the Co-HC, calcitriol reduced the frequency of polyclonally activated T cells expressing the activation markers HLA-DR and CD38, and those HLA-DR^+^CD38^-^, whereas increased the subpopulation HLA-DR^-^CD38^+^. Calcitriol treatment also decreased production of granzyme, IL-2 and MIP-1β by T cells and increased the transcriptional expression of the inhibitor of NF-kB and the antiviral genes cathelicidin (CAMP) and APOBEC3G in PBMCs from Co-HC.

**Conclusion:**

Our *in vitro* findings suggest that VitD treatment could reduce HIV-1 transmission through a specific modulation of the activation levels and function of T cells, and the production of antiviral factors. In conclusion, VitD remains as an interesting potential strategy to prevent HIV-1 transmission that should be further explored.

## Introduction

During sexual transmission of HIV-1, mucosal immune activation plays a crucial role. The presence of activated T cells is associated with increased susceptibility to infection [1], [2], [3]; in particular, activation phenotypes of CD4^+^ T cells co-expressing the markers HLA-DR and CD38 and HLA-DR^+^CD38^-^ cells are preferentially infected by HIV-1 [4], [5]. In contrast, low immune activation, particularly at the exposed mucosa [6], is a hallmark for individuals who remain seronegative, despite frequent exposure to the virus (HESNs) [6], [7], [8]. This quiescence phenotype is suggested as a protective mechanism against HIV-1 transmission and it is characterized by low expression of CD69, HLA-DR and CD38 on T cells [7], [9], [10], and reduced pro-inflammatory cytokine levels at genital sites [11], [12], [13]. Thus, compounds modulating immune activation, specifically at the mucosa level, could be potential tools to reduce HIV-1 susceptibility and prevent viral transmission.

Cholecalciferol is the inactive form of Vitamin D (VitD) that requires a series of hydroxylation reactions to become the active form known as calcitriol [14]. It binds the VitD receptor (VDR) and the retinoid X receptor (RXR) and acts as a transcription factor that modulates the expression of several genes with VitD response elements (VDREs) at their promoters [14]. This includes genes that encode molecules influencing the antiviral and anti-inflammatory responses, such as antimicrobial peptides [15], IL-10 [16], and the NF-kB inhibitor (IkBα) [17]. Therefore, it is through these actions that VitD might influence immune activation and thus HIV-1 transmission.

In support, higher plasma levels of VitD and a higher level of VDR mRNA transcripts have been observed in peripheral blood mononuclear cells (PBMCs) and the genital mucosa of a Colombian HESNs cohort, compared to that of healthy HIV unexposed controls [18]. The expression of the VDR correlates with that of the anti-inflammatory cytokine, IL-10, and with antiviral peptides like defensins and cathelicidin (CAMP), among others [18], [19]. Furthermore, in a previous study, we found that cholecalciferol treatment of PBMCs from a Colombian cohort of healthy non-exposed individuals modulated the activation levels of T cells [20] and their susceptibility to HIV-1 infection [4]. Nonetheless, in these studies we used a precursor form of the VitD for treating the cells, which is found only in low amounts in systemic circulation requiring higher concentrations to reach similar effects to calcitriol [21]. In addition, cholecalciferol activation depends on the cellular machinery, and on the presence of polymorphisms in genes encoding signaling molecules of the VitD pathway, such as hydroxylases. Thus, cholecalciferol itself has limited potential as a preventive strategy against HIV-1. In this regard, we decided to go further, exploring the *in vitro* role of the active form calcitriol on the susceptibility of CD4^+^ T cells to HIV-1 infection. *In vitro* infections were carried out in cells from Colombian healthy non-exposed individuals (Co-HC) and HESNs, and from Canadian healthy non-exposed individuals (Ca-HC), using X4- and R5-tropic HIV-1 strains, respectively. We also evaluated the calcitriol effects on activation and function of T cells; in addition, we explored the expression of viral co-receptors, and transcription of a panel of antiviral genes in the Colombian individuals.

## Methods

### Study population

Blood samples were obtained from 12 Co-HC and 8 HESNs from Medellin, Colombia; in addition, 7 Ca-HC from Winnipeg, Canada were included. Inclusion criteria for HESNs group were seronegative status at the time of enrollment with a history of unprotected sexual intercourse with HIV positive partners who had detectable viral loads with 12 or more unprotected sexual intercourse in at least 3 consecutive months within 1 year of study enrollment. This study was performed according to the Helsinki declaration (1975, revised in 2000), and approved by the bioethics board of the Universidad de Antioquia and the University of Manitoba.

### Cell cultures and calcitriol treatment

The PBMCs were isolated by a density gradient with Hystopaque reagent (Sigma-Aldrich, St. Louis, MO, USA) or Lymphoprep^™^ (Alere Technologies AS, Oslo, Norway). The PBMCs, obtained from healthy donors, were treated with two concentrations of calcitriol (1x10^-8^M and 5x10^-10^M) or ethanol at 1% (EtOH), as vehicle control (Sigma-Aldrich) for 24 hours at 37°C and 5% CO_2_. The PBMCs from HESNs were treated with only one concentration of calcitriol at 1x10^-9^M due to sample limitation. PBMCs of all recruited individuals were stimulated with phytohemagglutinin (PHA) (Sigma-Aldrich) (8ug/ml) and IL-2 (50 IU/mL) (Sigma) for 48 hours, before HIV-1 infection, maintaining supplementation with calcitriol.

### HIV-1 infection assay

Two million of the polyclonally activated PBMCs from all Colombian donors, calcitriol or EtOH treated, were infected with 13 ng of an X4-tropic HIV-1 p24 [obtained from H9-HTLV-IIIB cells (ATCC-CRL-8543)], in the presence of 10ug/mL of polybrene (Sigma-Aldrich). For Canadian donors, 5x10^5^ of activated PBMCs calcitriol or EtOH as a vehicle control treated were infected with 1 ng of an R5-tropic HIV-1 p24 (BAL), in presence of 8ug/mL of polybrene (Sigma-Aldrich) (Differences in the number of cells are due to the experiments to perform in each cohort, and differences in viral concentration used, depended on infectious capacity of each viral strain). The infection was performed by spinoculation for 2 hours according to previous reports [4], [22],. Cells were washed and cultured in their respective calcitriol- or EtOH-supplemented medium, at 37°C and 5% of CO_2_ for 72 hours, except for cytokines and secreted factors detection and mRNA expression in the Co-HC group, where cells were cultured only for 24 hours.

Viral infection of CD4^+^ T cells was evaluated by detection of intracellular p24 using flow cytometry. Briefly, PBMCs were extracellularly stained with anti-CD3-PeCy5 (eBioscience), and anti–CD4-APC (eBioscience), followed by intracellular staining with anti-p24, (Beckman Coulter) using the Foxp3 permeabilization kit (eBioscience). Non-infected PBMCs were used as control of p24 antibody specificity. Cells were washed, fixed and acquired on the FACSCanto-II or the Fortessa (Colombian cohort) or the LSRII (Canadian donors) flow cytometers; analysis was performed in the FACSDiva v.8.0.1 software. Frequency of p24^+^CD4^+^ T cells (S1 Fig) and Mean Fluorescence Intensity (MFI) of p24 in total CD4^+^ T cells was determined. We also evaluated p24 levels in supernatants by enzyme-linked immunosorbent assay (ELISA) using the “Lentivirus-associated p24” ELISA kit (Cell Biolabs, San Diego, CA) in the Colombian samples.

### Immune activation levels on T cells

The effect of calcitriol on the expression of the activation markers CD38 and HLA-DR was evaluated on CD4^+^ and CD8^+^ T cells from Co-HC and HESNs by flow cytometry 72 hours post-infection. Fluorochrome labelled antibodies anti-CD4-APC, anti-CD3-PeCy5, anti-CD8-efluor450, anti-HLA-DR-FITC and anti-CD38-PeCy7 (eBioscience, Santa Clara, CA, USA) were used. Four subpopulations of T cells were identified: HLA-DR^+^CD38^+^, HLA-DR^+^CD38^-^, HLA-DR^-^CD38^+^, and HLA-DR^-^CD38^-^ (S1 Fig).

### Functional profile of T cells

The functional profile response of T cells was evaluated by measuring production of cytokines and effector molecules by flow cytometry. Approximately 1x10^5^ of PBCMs treated with calcitriol or EtOH and infected with HIV-1 from Co-HC, were cultured for 24 hours, in presence of brefeldin (1ug/mL) and monensin (1ug/mL). Extracellular staining for CD4^+^ and CD8^+^ T cells was done, as previously described, and intracellular staining was performed using the antibodies anti-IL-2-FITC, anti-IFN-γ-PeCy7, anti-TNF-α-PerCp-Cy5.5, anti-MIP-1β-PE, anti-granzyme-FITC and anti-perforin-PE (eBioscience).

### Expression of viral co-receptor CXCR4 on CD4^+^ T cells

The expression of CXCR4 was determined on calcitriol or EtOH treated-CD4^+^ T cells from Co-HC, after 72 hours post-infection using flow cytometry, with the antibody anti-CD184-PE (BD).

### Gene transcriptional expression

The transcriptional expression of genes was evaluated in 5x10^5^ calcitriol or EtOH treated-PBCMs from Co-HC, before and after 24h of HIV-1 infection, by real-time PCR. Cells were stored in Trizol reagent (Thermo) at -80°C; RNA was extracted by Direct-zol^™^ RNA MiniPrep (Zymo), and cDNA was synthetized using the High-Capacity cDNA Reverse Transcription Kit (ThermoFisher Scientific, St. Leon-Rot, Germany). Transcriptional expression of antiviral or VitD pathway genes was evaluated (S1 Table). The reported amount was relative to the expression of the reference genes PGK1 and β-actin, using the delta CT method.

### Prediction of Vitamin D response elements (VDRE) on modulated genes

The predicted presence of VDRE was searched on promotor regions of genes that were modulated by calcitriol, including activation markers, cytokines, and effector molecules and antiviral peptides, using the Jasper software (http://jaspar.genereg.net/cgi-bin/jaspar_db.pl) (S2 Table).

### Statistical analysis

Data were analyzed on the GraphPad Prism v.7.05 software. Parametric or non-parametric tests were applied according to the normality of data. Ratio T test or Wilcoxon test were used to compare the differences between the EtOH control and the calcitriol treatments. Results are presented as median or mean and p-value <0.05 was considered statistically significant.

## Results

### Demographic data

In the HESNs group, the average length of a sexual relation with an HIV-1 infected partner was 83 months, with a frequency of 14 intercourses monthly during the time of exposure, in which a detectable viral load of the seropositive partner was confirmed. Demographic data is shown in Table 1.

**Table 1:** Demographic data of Colombian individuals.

	Length of exposure (months)	Monthly frequency (times)	Viral Load (SP partner) (Median; Range) copies/mL	CD4 count (SP partner). (Median; Range) Cells/ul	Gender (F/M)*
HESN	83	14	1200 (<10–180790)	344 (134–804)	6/2
Non-exposed	-	-	--		4/8

*F = female; M = male

### Calcitriol reduced the infection of CD4^+^ T cells

**Fig 1:**
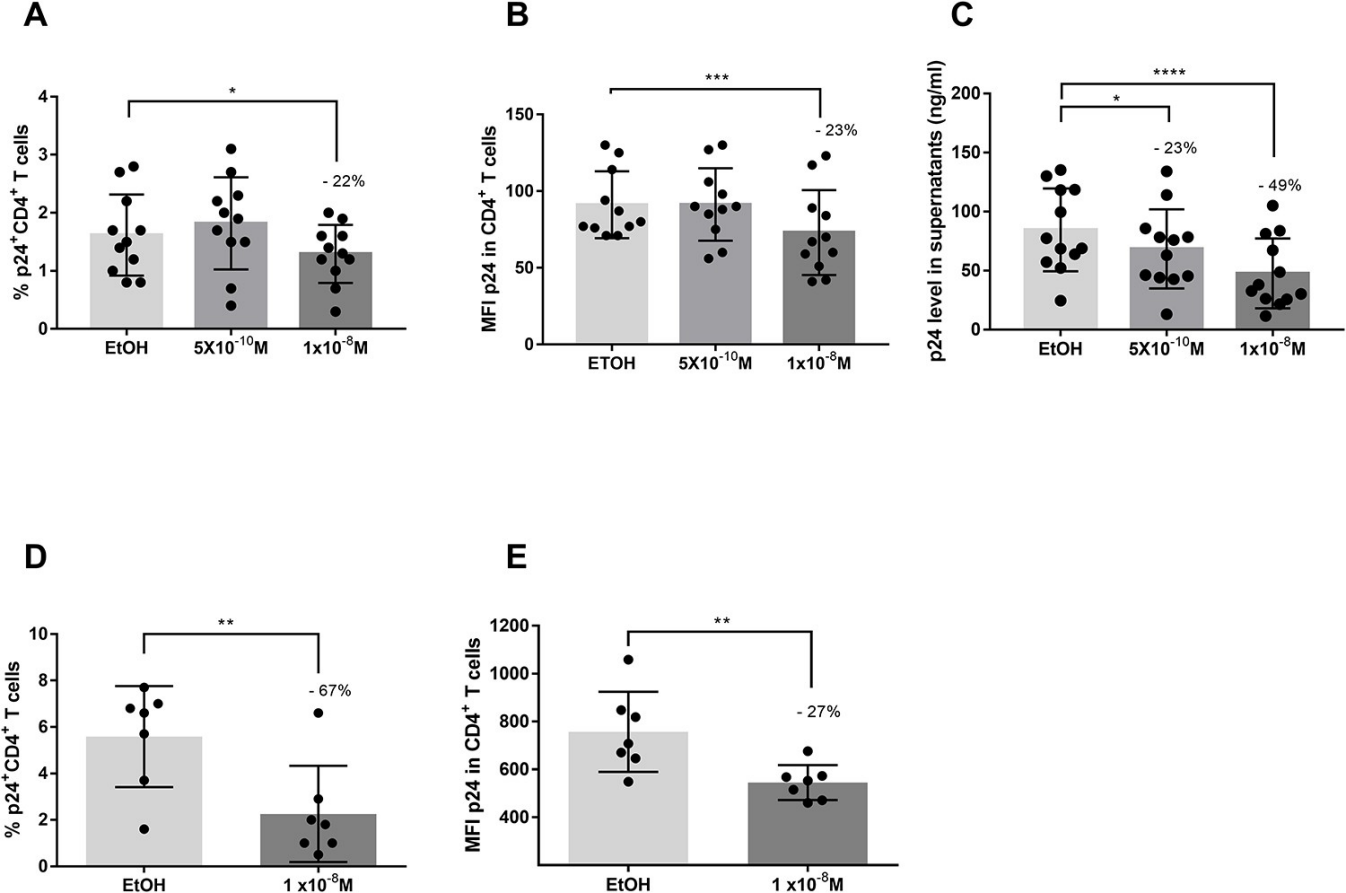
HIV-1 infection of CD4^+^ T cells from non-exposed donors after treatment with calcitriol. Percentage of p24^+^CD4^+^ T cells (**A**), Mean Fluorescence Intensity (MFI) of p24 in total CD4^+^ T (**B**) and Levels of p24 in supernatants (**C**), after treatment of PBMCs from the Colombian non-exposed group with calcitriol at 1x10^-8^M and at 5x10^-10^M or EtOH and infection with an X4-tropic HIV-1 (n = 12). Percentage of p24^+^CD4^+^ T cells (**D**) and MFI of p24 in total CD4^+^ T (**E**), after treatment of PBMCs from the Canadian non-exposed group with calcitriol at 1x10^-8^M or EtOH and infection with an R5-tropic HIV-1 (n = 7). Comparison between EtOH and VitD treatments were made using the Ratio paired t-test, (*) p≤ 0.05; (**) p≤ 0.01; (***) p≤ 0.001; (****) p≤ 0.0001. The percentage of reduction compared to EtOH is showed in each figure.

To determine the effect of VitD on the susceptibility to HIV-1 infection of CD4^+^ T cells, PBMCs pre-treated with calcitriol from Co-HC and Ca-HC were infected with an X4- or an R5-tropic virus, respectively. The treatment with the higher concentration of calcitriol, decreased the percentage of infected CD4^+^ T cells with the X4-tropic HIV-1 by 22%, (p = 0.0446) (Fig 1A), and reduced the MFI of p24 in CD4^+^ T cells by 23% (p = 0.0009) (Fig 1B), compared to EtOH. Both concentrations of VitD, decreased the released viral particles in supernatants in a dose-dependent manner by 23% up to 49% compared to the control (p<0.0001 and p = 0.0465) (Fig 1C).

Furthermore, calcitriol treatment exhibited a more potent effect on decreasing infection of CD4^+^ T cells with a more infective R5 (Bal) HIV-1 strain in our Canadian cohort, with a reduction between 57% and 67% observed for both concentrations of this hormone, compared to EtOH (p = 0.0018 and p = 0.0068) (Fig 1D). Similarly, the MFI of p24 in CD4^+^ T cells was decreased by 15% and 27%, in comparison to EtOH (p = 0.0046 and p = 0.0177) (Fig 1E). Thus, VitD exhibited an *in vitro* protective role against HIV-1 infection, decreasing the susceptibility of CD4^+^ T, regardless the viral strain.

### Calcitriol modulated the activation phenotype of T lymphocytes

**Fig 2:**
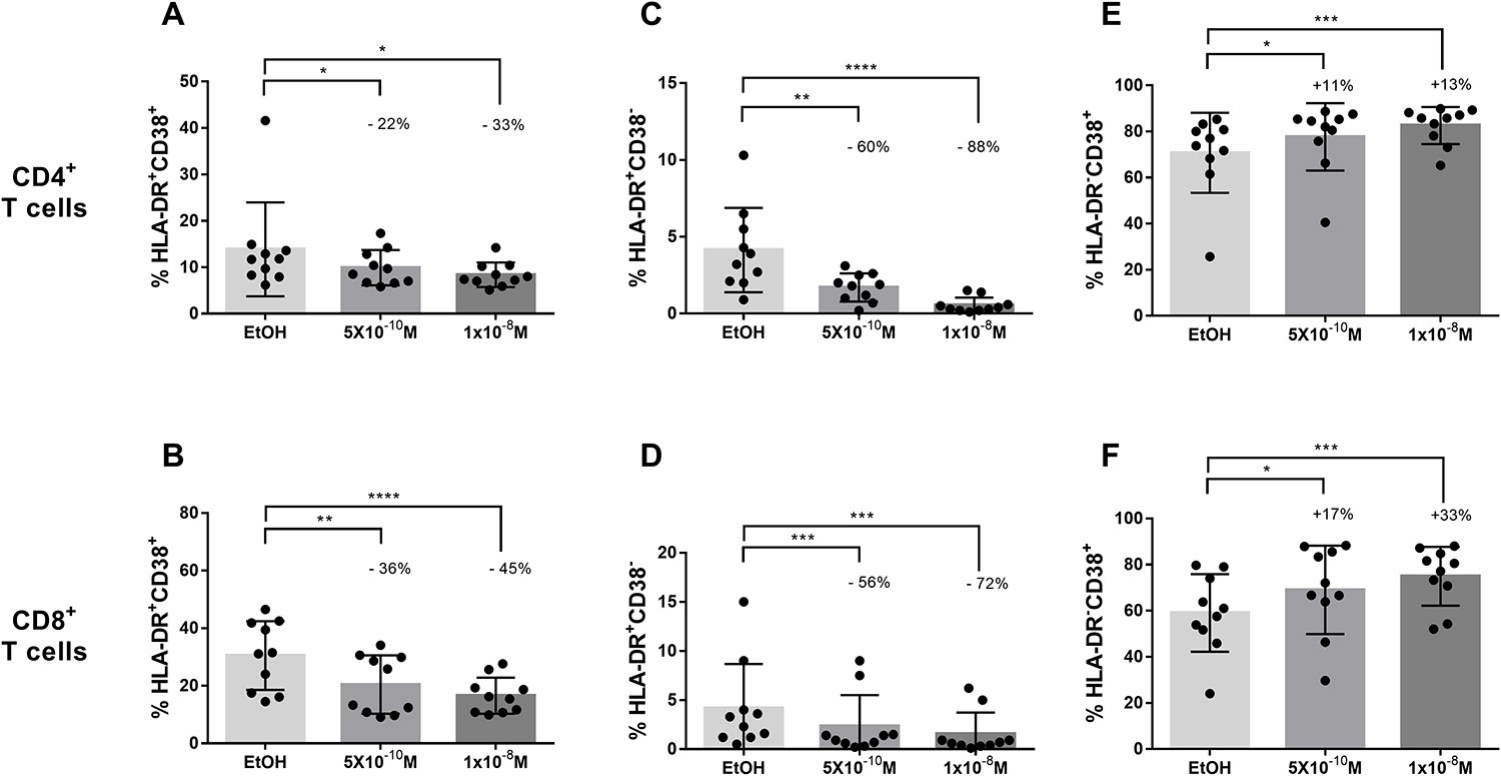
Subpopulations of activated CD4^+^ and CD8^+^ T cells according to surface expression of activation markers HLA-DR and CD38, after calcitriol treatment of PBMCs from the Colombian non-exposed group (n = 10). Percentage of HLA-DR^+^CD38^+^ in CD4^+^ (**A**) and CD8^+^ (**B**) T cells. Percentage of HLA-DR^+^CD38^-^ in CD4^+^ (**C**) and CD8^+^ (**D**) T cells. Percentage of HLA-DR^-^CD38^+^ in CD4^+^ (**E**) and CD8^+^ (**F**) T cells. Comparison between treatments were made using the Ratio paired t-test, (*) p≤ 0.05; (**) p≤ 0.01; (***) p≤ 0.001; (****) p≤ 0.0001. The percentage of reduction (-) or increase (+) compared to EtOH is showed in each figure.

Given the importance of immune activation in the susceptibility to HIV-1 infection, we determined the effect of calcitriol on expression of HLA-DR and CD38 activation markers in polyclonally-activated T cells from the Co-HC. Both concentrations of calcitriol significantly decreased the percentage of CD4^+^ T cell co-expressing HLA-DR and CD38 by 22% up to 33%, compared to EtOH (p = 0.0137 and p = 0.0131) (Fig 2A); such effect was found more evident in CD8^+^ T cells with a reduction between 36% and 45% (p<0.0001 and p = 0.0010) (Fig 2B).

Calcitriol also decreased the frequency of HLA-DR^+^CD38^-^ in CD4^+^ T cells by about 60% to 88% compared to EtOH (p<0.0001 and p = 0.0010) (Fig 2C) and, in CD8^+^ T cells by 56% up to 72% (p = 0.0002 and p = 0.001) (Fig 2D). In contrast, HLA-DR^-^CD38^+^ cells were increased with both calcitriol concentrations, in CD4^+^ T cells by 11% and 13% (p = 0.001 and p = 0.0322) (Fig 2E) and in CD8^+^ T cells by 17% and 33% (p = 0.001 and p = 0.0186) (Fig 2F).

In addition, the treatment also augmented in a dose dependent manner, the MFI of CD38 in CD4^+^ between 34% and 185% (p<0.0001 and p<0.0001) (S2A Fig) and in CD8^+^ T cells by 36% up to 173% (p<0.0001 and p<0.0001) (S2B Fig); whereas for HLA-DR, a reduction was observed in CD8^+^ by 31% and 20% (p = 0.0068 and p = 0.0021) (S2C Fig) but not in CD4^+^ T cells (S2D Fig).

Interestingly, both CD38 and HLA-DR genes exhibit four and three VDREs at their promoter regions respectively (S2 Table).

In this regard, VitD exerted a selective modulation on the activation of T cells that might be partially responsible for the reduced susceptibility to HIV-1 infection.

Although, we evaluated the expression of the viral coreceptor CXCR4 after calcitriol treatment in Co-HC, there was not effect neither on the frequency of CD4^+^ T cells expressing CXCR4 nor the MFI of CXCR4 (S3A and S3B Fig) despite the presence of 2 VDREs downstream to the transcription start site (TSS) of this gene.

### Calcitriol regulates functional profile response of T cells

**Fig 3:**
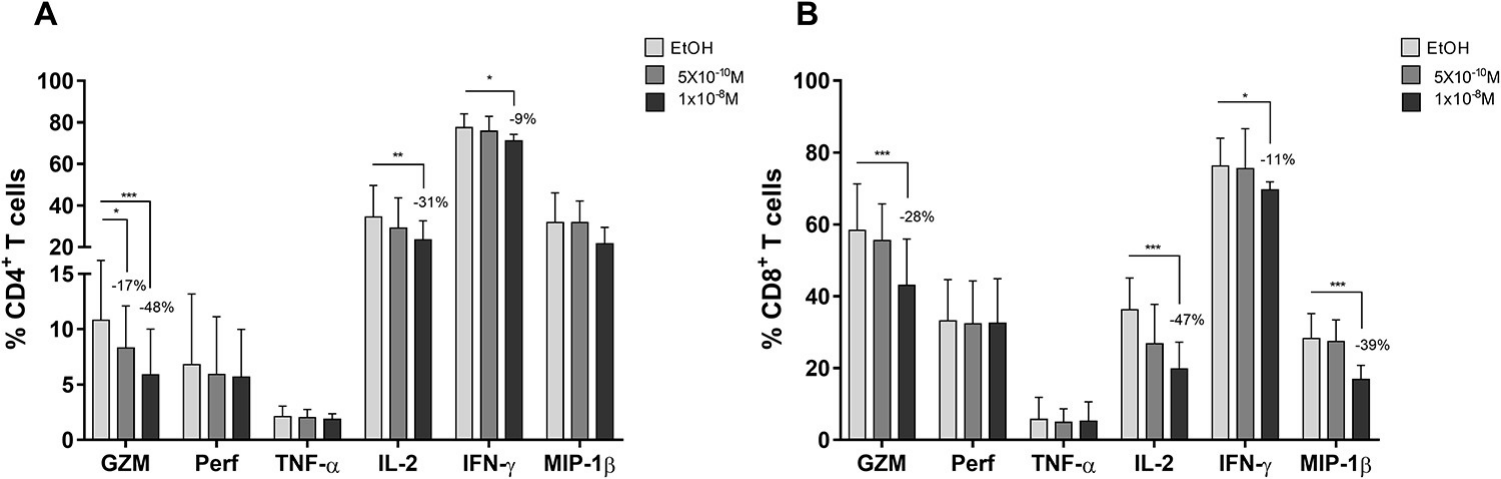
Frequency of CD4^+^ (A) and CD8^+^ (B) T cells producing cytokines and effector molecules after calcitriol treatment of PBMCs from the Colombian non-exposed group (GZM n = 12; Perf n = 12; TNF-α n = 7; IL2 n = 9; IFN-γ n = 7; Mip-1β n = 11). Differences among the n are due to difficulties for the analysis of some cytokines. Comparison between treatments were made using the Ratio paired t-test, (*) p≤ 0.05; (**) p≤ 0.01; (***) p≤ 0.001. The percentage of reduction compared to EtOH is showed in each figure.

The effect of VitD on T cells response, in terms of cytokines and effector molecules production was also evaluated in cells from Co-HC. In CD4^+^ T cells, calcitriol decreased the frequency of polyclonally-activated cells producing granzyme (GZM), in a dose dependent manner by 17% to 48% (p = 0.0001 and p = 0.0308); also, the higher concentration of this hormone significantly reduced the production of IL-2 by 31% (p = 0.0091) and IFN-γ by 9% (p = 0.0299) compared to EtOH. However, the treatment did not affect the production of the other evaluated molecules, perforin, TNF-α and MIP-1β (Fig 3A).

In CD8^+^ T cells, the higher concentration of calcitriol also decreased the frequency of cells producing GZM by 28% (p = 0.0005), IL-2 by 47% (p = 0.0007), IFN-γ by 11% (p = 0.0125) and MIP-1β by 39% (p = 0.0007); whereas the production of the remaining molecules, perforin, TNF-α, was not altered (Fig 3B). For both GZM and IL-2 genes we found one VDRE at their promoter region and downstream to TSS, respectively.

Thus, VitD further than modulate the activation of T cells, it also regulated the functional profile of response of these cells.

### Calcitriol treatment increased the expression of the antiviral and anti-inflammatory genes

**Fig 4:**
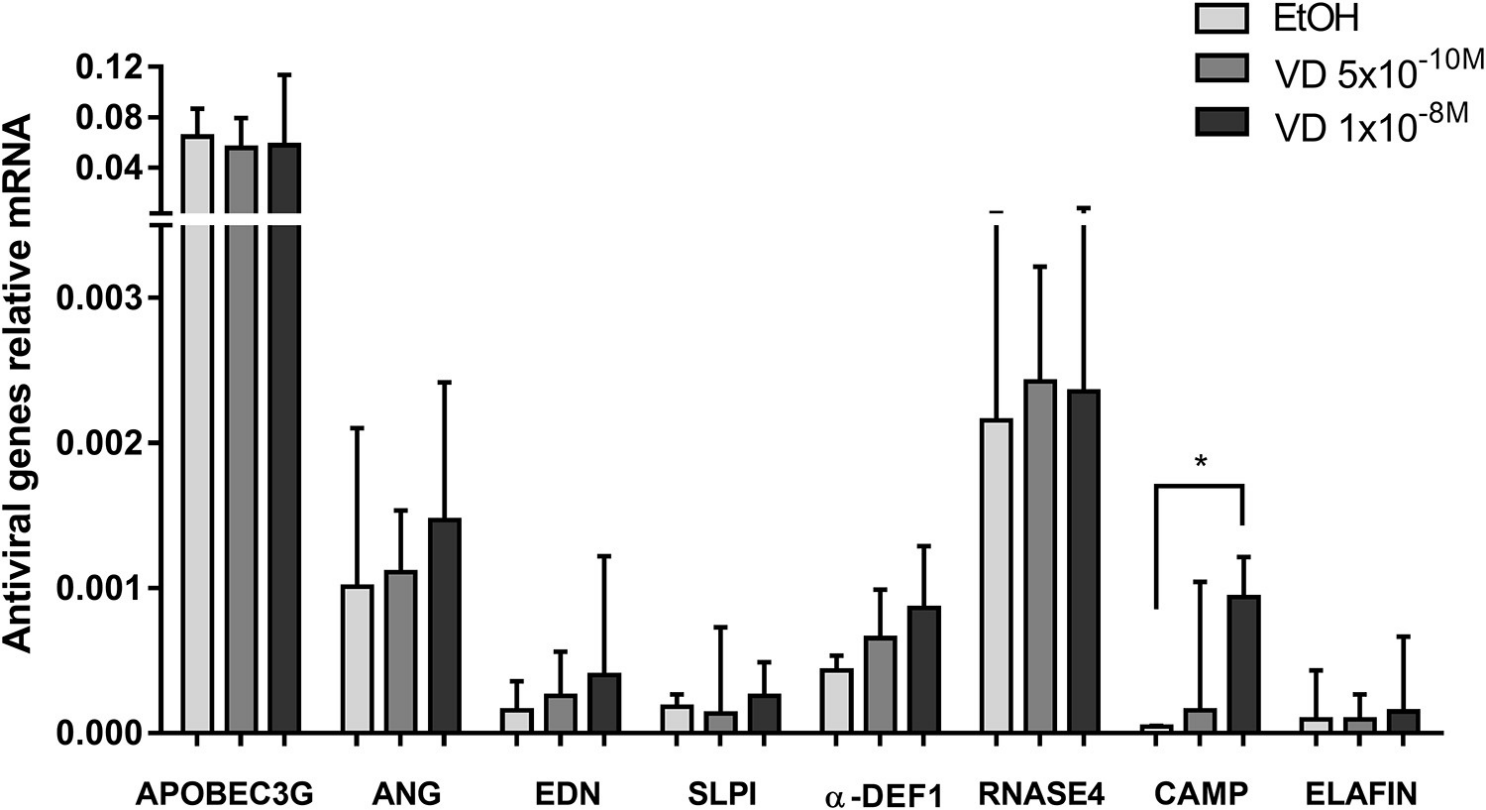
Relative expression of antiviral genes in PBMC from the Colombian non-exposed group, after treatment with calcitriol (APOBEC3G n = 12; ANG n = 11; END n = 11; SLPI n = 8; α-DEF1 n = 5; RNAse4 n = 10; CAMP n = 6; Elafin n = 10). Relative expression normalized to PGK1 and β-actin genes. Differences among the n are due to the lack of amplification of some genes in certain healthy donors. Comparisons for each antiviral gene expression in presence of EtOH or calcitriol were made using the Wilcoxon test given non-normality of data.

In addition, we evaluated the effect of calcitriol treatment on the transcriptional expression of several genes related to antiviral and anti-inflammatory responses and the VitD pathway, in PBMCs from Co-HC, in absence or presence of HIV-1 infection. As expected, the higher concentration of calcitriol induced the expression of the hydroxylase, CYP24A1 (p = 0.0001). It also increased the expression of the inhibitor IKBα (p = 0.0228) and the antiviral gene CAMP (p = 0.0007) that has one VDRE at its promoter region, but not EDN (p = 0.0597) or SLPI (p = 0.0697) before HIV-1 infection (Fig 4A). Moreover, twenty-four hours post-infection, calcitriol, at 5x10^-10^M, increased the mRNA levels of APOBEC-3G (p = 0.0116) (**Data not shown**). The expression of the remaining genes was not affected by calcitriol treatment. In this regard, VitD might also protect against HIV-1 infection through induction of an antiviral response mainly mediated by CAMP and APOBEC3G molecules.

### Calcitriol treatment did not affect the infection of CD4^+^ T cells in HESNs

**Fig 5:**
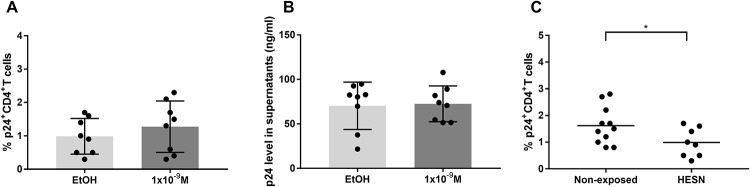
HIV-1 infection of CD4^+^ T cells from HESNs after treatment with calcitriol. Percentage of p24^+^CD4^+^ T cells (**A**) and levels of p24 in supernatants (**B**), after treatment of PBMCs from the Colombian HESNs with calcitriol at 1x10^-9^M or EtOH and infection with an X4-tropic HIV-1 (n = 8). Comparison between treatments were made using the Ratio paired t-test. Percentage of p24^+^CD4^+^ T cells in untreated PBMCs (EtOH) from Colombian HESNs (n = 8) and non-exposed donors (n = 11) (**C**). Comparison were made by two-tailed, Mann-Whitney test, (*) p≤0.05.

As calcitriol exhibited a protective effect on HIV-1 susceptibility from non-exposed donors from Colombia and Canada, we evaluated such effect in HESNs. For this, we used an intermediate concentration of 1x10^-9^M due to sample limitation. In contrast to the results observed in non-exposed individuals, calcitriol did not affect the percentage of X4-tropic HIV-1 infected CD4^+^ T cells in HESNs (Fig 5A) neither the MFI for p24 in CD4^+^ T cells. The amount of released viral particles was similar in calcitriol and EtOH treatments (Fig 5B).

Considering these results, we evaluated differences in the susceptibility to HIV-1 infection between Co-HC and HESNs in absence of calcitriol treatment. As previously described, we observed a lower percentage of infected CD4^+^ T cells in the HESNs group (0.95% vs. 1.5%, p = 0.0239) (Fig 5C).

### Particular modulation of T cells activation exerted by calcitriol in HESNs

Contrasting with our observations for Co-HC, calcitriol treatment of cells from HESNs reduced the frequency of HLA-DR^+^CD38^+^CD4^+^ T cell by 22.4% compared to EtOH (p = 0.0021) (S4A Fig) but not for CD8^+^ T cell (S4B Fig). The frequency of HLA-DR^+^CD38^-^CD4^+^ and CD8^+^ were also decreased by 62.1% (p = 0.0017) (S4C Fig) and 37.1% (p = 0.0094) (S4D Fig) respectively, compared to EtOH; whereas the subpopulation HLA-DR^-^CD38^+^ was increased only in CD8^+^ by 17.5% (p = 0.0159) (S4F Fig) but not in CD4^+^ T cells (S4E Fig).

Given the discrepancy in modulation observed by calcitriol between non-exposed individuals and HESNs, we compared the frequency of the activated subpopulations of T cells in both groups of individuals in absence of calcitriol treatment and after polyclonal stimuli. For CD4^+^ T cells, we did not observed differences in HLA-DR^+^CD38^+^ (S5A Fig) nor HLA-DR^-^CD38^+^ cells (S5B Fig), whereas in CD8^+^ T cells, HLA-DR^+^CD38^+^ were significantly higher in HESNs (55.4% vs. 30.5%, p = 0.0023) (S5C Fig) while HLA-DR^-^CD38^+^ were lower (31.9% vs. 59.1%, p = 0.0027) (S5D Fig) compared to non-exposed individuals; these findings suggest an inherent differential phenotype of activation and potentially, of response upon challenge, between HESNs and non-exposed individuals.

## Discussion

During sexual exposure to HIV-1 infection, mucosal CD4^+^ T cells play a critical role in transmission, particularly, activated cells, as activation favors productive infection [2], [1]; In contrast, the immune quiescent phenotype described at genital mucosa of HESN [11], [12], [13] is associated with reduced susceptibility to infection. Considering this, current approaches are focused on the use of compounds that can modulate immune responses prior to and/or triggered by the viral encounter to reduce HIV-1 transmission.

VitD through its modulatory effects on the immune system [15], [16], [17] contributes to maintain an anti-inflammatory environment and enhanced antiviral activity that might reduce HIV-1 transmission. Indeed, higher plasma levels of VitD and VDR mRNA expression are observed in PBMCs and the genital mucosa from HESNs compared to non-exposed healthy donors [18], that are correlated with the expression of the anti-inflammatory cytokine IL-10. This elevated IL-10 was associated with increased expression of the antiviral genes β-defensins, CAMP, Elafin, RNAse7 and TRIM5 in genital mucosa [18], [19].

In this *in vitro* study, calcitriol induced a protective effect reducing HIV-1 infection of CD4^+^ T from non-exposed healthy individuals, irrespectively of the X4- or R5-viral tropism. These results are in agreement with our previous findings using the inactive form cholecalciferol [4] and with studies of oral VitD supplementation of healthy individuals that reduces HIV-1 infection of PBMCs [23]. Taken together, these findings highlight the potential of VitD supplementation to become a cost-effective preventive strategy against HIV-1 transmission. However, some *in vitro* studies reported a contrasting increase of HIV-1 replication in a monocytic cell line after calcitriol treatment that should be taken into account [24], [25], [26].

The reduced HIV-1 infection observed in the presence of calcitriol is likely related to the modulation of T cells activation induced by this hormone, as a decreased frequency of subpopulations co-expressing HLA-DR and CD38 and those HLA-DR^+^CD38^-^ was observed, similar to our previous findings for the precursor cholecalciferol [20]. In the case of CD4^+^ T cells, both activated subpopulations exhibit a higher susceptibility to infection [4], [5], whereas for CD8^+^ T cells, high frequency of HLA-DR^+^CD38^-^ is associated with seroconversion during continued HIV-1 exposure [27], suggesting that by reducing the frequency of these different cells, VitD might decrease the HIV-1 infection risk.

In parallel, calcitriol increased cells expressing exclusively CD38 that exhibit lower susceptibility to HIV-1 infection [4], [28], reduced proliferation and increased production of IL-2 and IFN-γ [29]. Further, the CD38 molecule resembles a homologous sequence of the Loop V3 region of gp120 that interacts with the CD4 molecule blocking the access of the virus to this receptor [28], [30], [31]; and indeed, a soluble form of CD38 is being studied as a potential strategy against this viral infection [31]. Thus, augmented CD38 expression induced by calcitriol might have a role in reducing HIV-1 infection of CD4^+^ T cells.

Interestingly, both activation markers, HLA-DR and CD38, have four and three VDREs at their promoter regions respectively, suggesting that the effect of VitD inducing or reducing their expression depends on the specific gene; nonetheless, the reasons explaining such a contrasting effect remain to be determined.

Along with the modulation of immune activation, calcitriol also decreased some of the effector functions of polyclonally activated T cells such as the production of granzyme B, IL-2, IFN-γ and MIP-1β; according to our results, other authors have reported a similar effect [32], [33], [34], [35]. Although for TNF-α and perforin we did not observe an effect by calcitriol treatment, contrasting reports have shown decreased expression of these molecules [36], [37]. Indeed, VitD also reduced production of other proinflammatory cytokines favoring an anti-inflammatory environment [38], suggesting that maintaining a regulated state of immune activation is crucial for preventing HIV-1 infection, rather than a potent initial immune response during viral exposure.

Calcitriol also induced the expression of CYP24A1 and IkBα genes, and the antiviral genes CAMP and APOBEC3G; perhaps unsurprisingly as all these genes have VDREs at their promoter regions. The augmented expression of CYP24A1 confirmed that the PBCMs from our individuals experienced increased intracellular levels of calcitriol, contrasting to our previous results using the precursor calcidiol, where this hydroxylase was not increased [4], and suggesting that calcitriol treatment might be more efficient to trigger VitD modulated pathways. Similar to our results, other reports have also shown induction of IkBα that is related to the inhibition of the NF-kB signaling pathway, and thus the inflammatory response [17], [39], [40], contributing to support our hypothesis of a regulated environment triggered by VitD that might reduce HIV-1 infection.

According to the increased expression of antiviral genes, other authors observed increased plasma levels of CAMP after VitD supplementation of healthy donors and HIV-1 infected individuals [41], and induced expression of APOBEC3G by *in vitro* treatment of cells [4].

Unexpectedly, calcitriol treatment did not reduce the infection of CD4^+^ T cells from HESNs that could be explained by several reasons, including the differential modulation exerted by this hormone on the activation state of T cells from HESNs compared to non-exposed. In addition, higher pre-existing levels of plasma VitD as previously described by us in a similar cohort [18], although for these individuals such levels were not determined due to samples limitation; and/or presence of a pre-established protective phenotype through other resistance mechanisms triggered *in vivo* in the context of the frequent viral exposure. Indeed, since our HESNs exhibited a lower susceptibility of infection compared to non-exposed individuals, the responsible mechanism(s) for resistance are maintained even during an *in vitro* challenge.

Certainly, the differential activation profile of T cells in response to the polyclonal stimuli observed between HESNs and non-exposed, with increased frequency of HLA-DR^+^CD38^+^ and lower HLA-DR^-^CD38^+^CD8^+^ T cells could potentially contribute to explain, at least partially, for the differences in susceptibility to infection.

In terms of a global strategy to utilize VitD as a potential prophylactic treatment, the use of calcitriol instead of its precursors could be a better strategy. This is because the precursors transformation into calcitriol may be affected by the presence of genetic polymorphisms altering hydroxylases production and therefore, impacting its bioavailability and effects on the immune system according to the individuals genetic background [23]. Furthermore, calcitriol exhibited a 100-fold high potent activity than calcidiol and a consistent effect on regulating VDR target gene expression [42].

To highlight, since we used PBMCs for all our experiments, an effect of calcitriol on other cells like monocytes, B cells, NK cells or others, that could be influencing our findings on T cells cannot be ruled out. In addition, since we used an acute treatment with EtOH at 1% as a control of our experiments, it could have influenced our results; indeed, EtOH has shown to affect the function of monocytes favoring a regulatory profile [43], [44], [45], whereas in T and B lymphocytes might induce apoptosis [45].

Finally, our findings suggest that treatment with calcitriol might be a potential and cost-effective strategy to reduce the incidence of HIV-1 infection worldwide, through the regulation of the immune activation and induction of an antiviral state.

## Supporting information

S1 Fig Gating strategy to define the cell populations by flow cytometry. The analysis of data was performed using the FacsDiva v.8.0.1 software. Aggregates exclusion and lymphocyte region were defined according to FSC and SSC parameters. The frequency of infected cells, p24^+^CD4^+^ T cells was defined from the CD3^+^ and CD4^+^ gate (**A**). The expression of activation markers, CD38 and HLA-DR was evaluated on CD4+ (**B**) and CD8+ (**C**) T cells.
(TIF)

S2 Fig Mean Fluorescence Intensity (MFI) for CD38 and HLA-DR in T cells after calcitriol treatment of PBMCs from the Colombian non-exposed group. MFI of CD38 in CD4^+^ (**A**) and CD8^+^ (**B**) T cells. MFI of HLA-DR in CD8^+^ (**C**) and CD4^+^ (**D**) T cells (n = 8). Comparison between treatments were made using the Ratio paired t-test, (*) p≤ 0.05; (**) p≤ 0.01; (***) p≤ 0.001; (****) p≤ 0.0001. The percentage of reduction (-) or increase (+) compared to EtOH is showed in each figure.
(TIF)

S3 Fig Frequency of CD4^+^ T cells expressing the viral coreceptor CXCR4 (A) and MFI of CXCR4 in CD4^+^ T cells (B) after calcitriol treatment of PBMCs from the Colombian non-exposed group (n = 12). Comparisons between EtOH and calcitriol were made using the Ratio paired t-test.
(TIF)

S4 Fig Subpopulations of activated CD4^+^ and CD8^+^ T cells according to the surface expression of activation markers HLA-DR and CD38, after calcitriol treatment (1x10^-9^M) of PBMCs from the Colombian HESNs (n = 7). Percentage of HLA-DR^+^CD38^+^ in CD4^+^ (**A**) and CD8^+^ (**B**) T cells. Percentage of HLA-DR^+^CD38^-^ in CD4^+^ (**C**) and CD8^+^ (**D**) T cells. Percentage of HLA-DR^-^CD38^+^ in CD4^+^ (**E**) and CD8^+^ (**F**) T cells. Comparison between treatments were made using the Ratio paired t-test, (*) p≤ 0.05; (**) p≤ 0.01. The percentage of reduction (-) or increase (+) compared to EtOH is showed in each figure.
(TIF)

S5 Fig Comparison of activated CD4^+^ and CD8^+^ T cells according to surface expression of activation markers HLA-DR and CD38, between untreated PBMCs from the Colombian HESNs (n = 7) and non-exposed donors (n = 10). Percentage of HLA-DR^+^CD38^+^ in CD4^+^ (**A**) and CD8^+^ (**B**) T cells. Percentage of HLA-DR^+^CD38^-^ in CD4^+^ (**C**) and CD8^+^ (**D**) T cells. Comparison between groups were made by two-tailed, Mann-Whitney test, (*) p≤ 0.05; (**) p≤ 0.01.
(TIF)

S1 Table Primers sequences for genes evaluated after treatment of PBMCs with calcitriol. (DOCX)

S2 Table Presence of VDREs at the specific genes evaluated by Jasper software. (DOCX)
