# Supplementary figures and images for: Vitamin D treatment of peripheral blood mononuclear cells modulated immune activation and reduced susceptibility to HIV-1 infection of CD4+ T lymphocytes
Source: PLoS One. 2019 Sep 24;14(9):e0222878. doi: 10.1371/journal.pone.0222878 (PMC6759150; doi:10.1371/journal.pone.0222878)

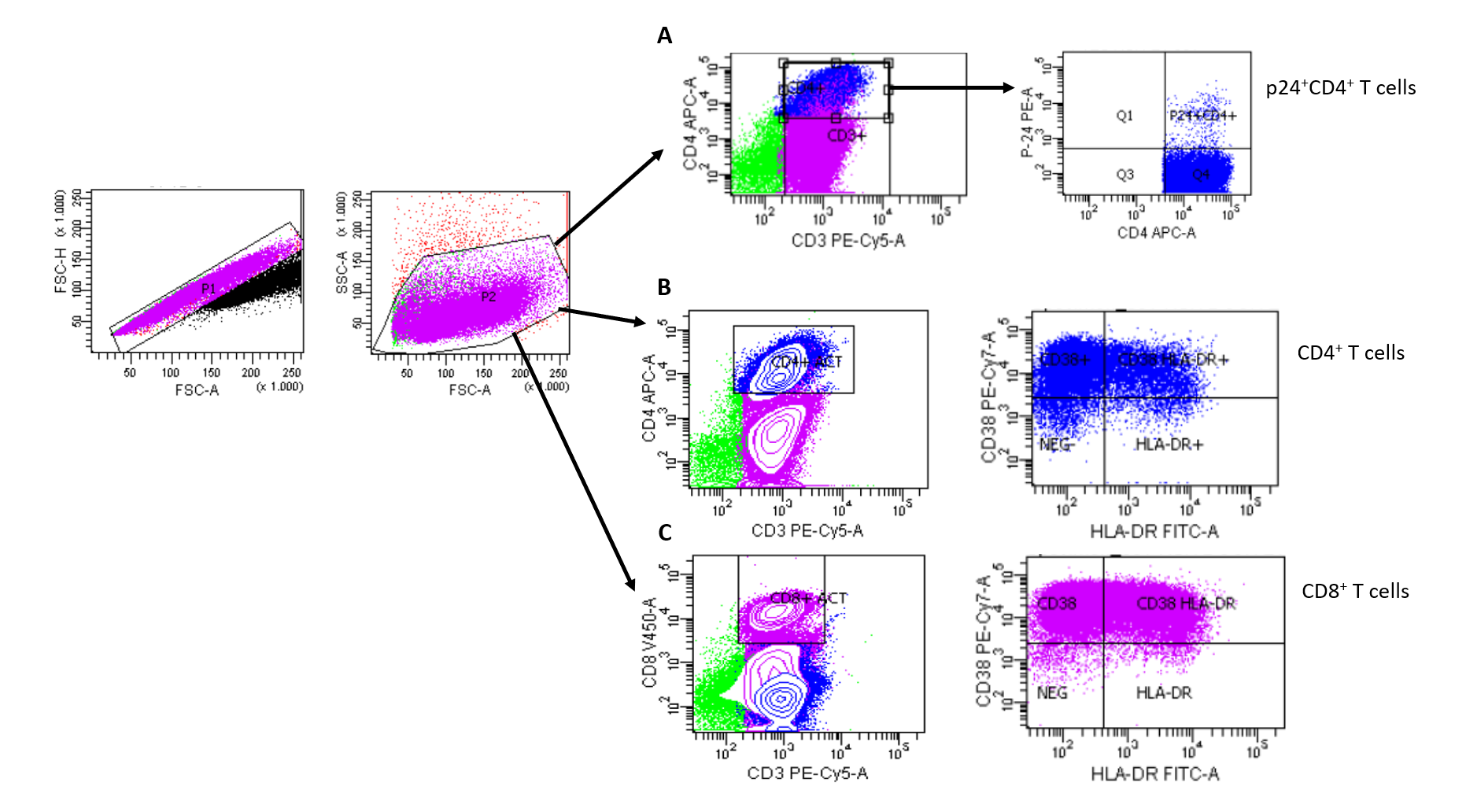

Supplement: S1 Fig — The analysis of data was performed using the FacsDiva v.8.0.1 software. Aggregates exclusion and lymphocyte region were defined according to FSC and SSC parameters. The frequency of infected cells, p24+CD4+ T cells was defined from the CD3+ and CD4+ gate (A). The expression of activation markers, CD38 and HLA-DR was evaluated on CD4+ (B) and CD8+ (C) T cells. (TIF) [file pone.0222878.s001.tif]

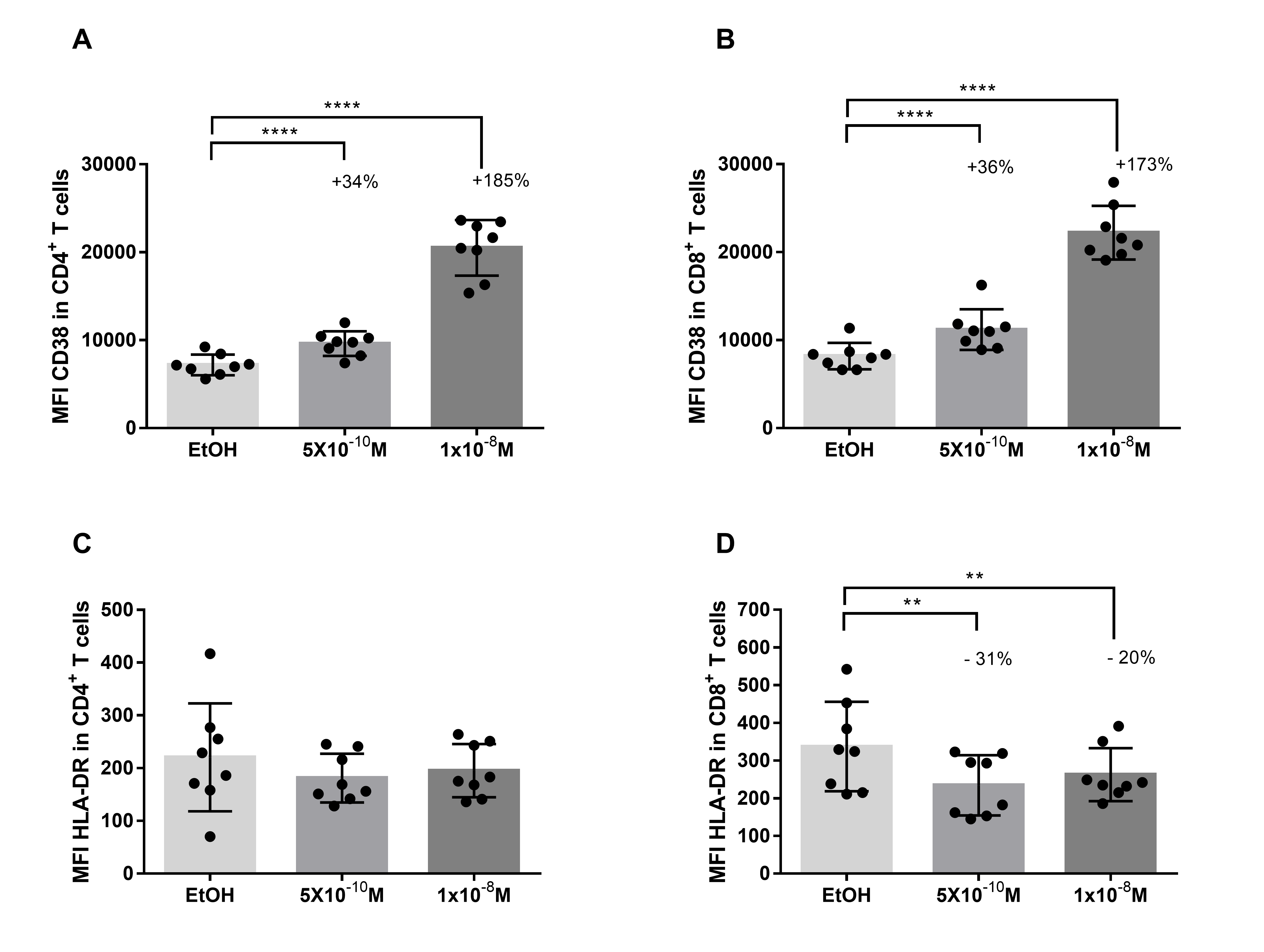

Supplement: S2 Fig — MFI of CD38 in CD4+ (A) and CD8+ (B) T cells. MFI of HLA-DR in CD8+ (C) and CD4+ (D) T cells (n = 8). Comparison between treatments were made using the Ratio paired t-test, (*) p≤ 0.05; (**) p≤ 0.01; (***) p≤ 0.001; (****) p≤ 0.0001. The percentage of reduction (-) or increase (+) compared to EtOH is showed in each figure. (TIF) [file pone.0222878.s002.tif]

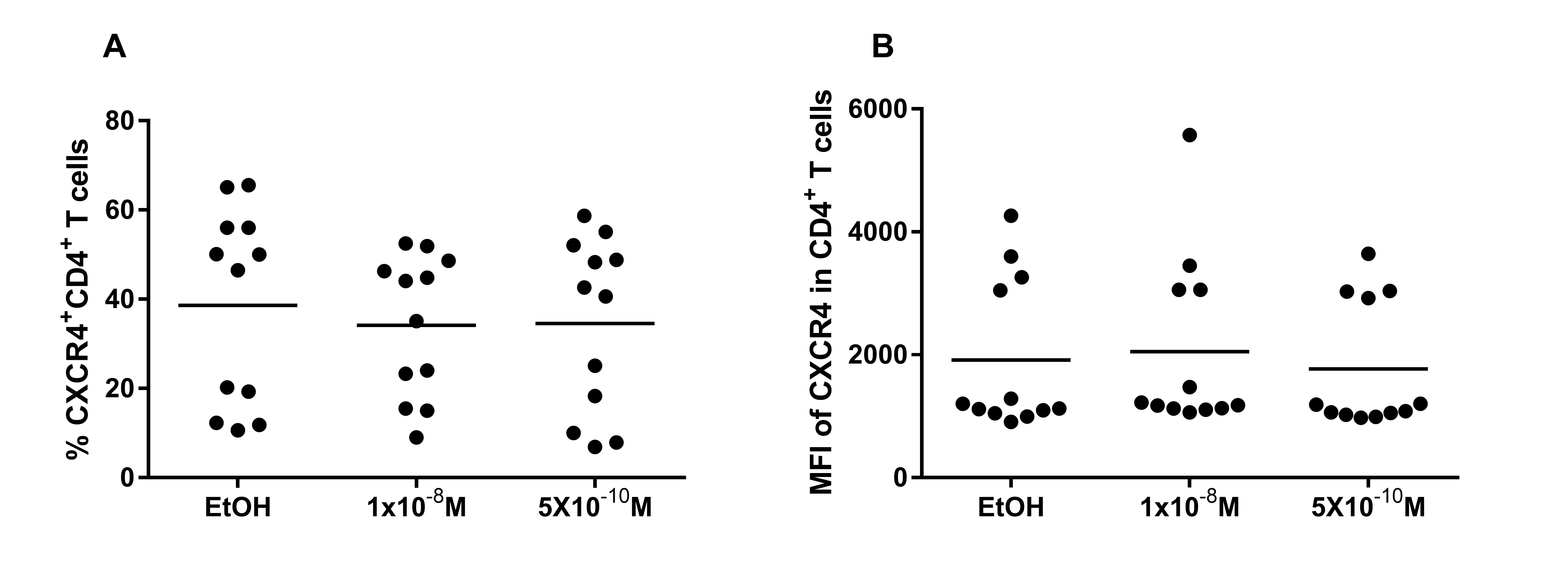

Supplement: S3 Fig — Comparisons between EtOH and calcitriol were made using the Ratio paired t-test. (TIF) [file pone.0222878.s003.tif]

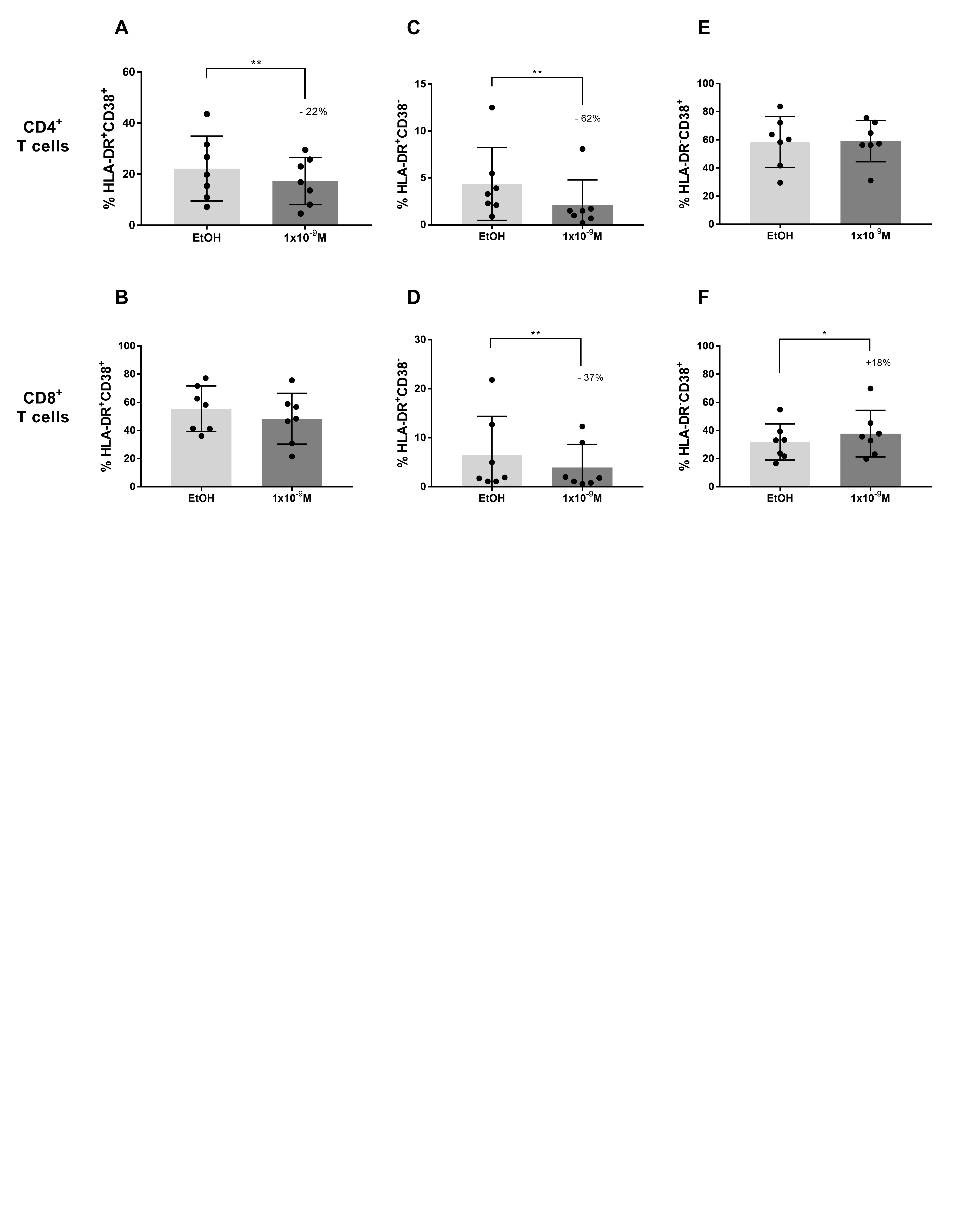

Supplement: S4 Fig — Percentage of HLA-DR+CD38+ in CD4+ (A) and CD8+ (B) T cells. Percentage of HLA-DR+CD38- in CD4+ (C) and CD8+ (D) T cells. Percentage of HLA-DR-CD38+ in CD4+ (E) and CD8+ (F) T cells. Comparison between treatments were made using the Ratio paired t-test, (*) p≤ 0.05; (**) p≤ 0.01. The percentage of reduction (-) or increase (+) compared to EtOH is showed in each figure. (TIF) [file pone.0222878.s004.tif]

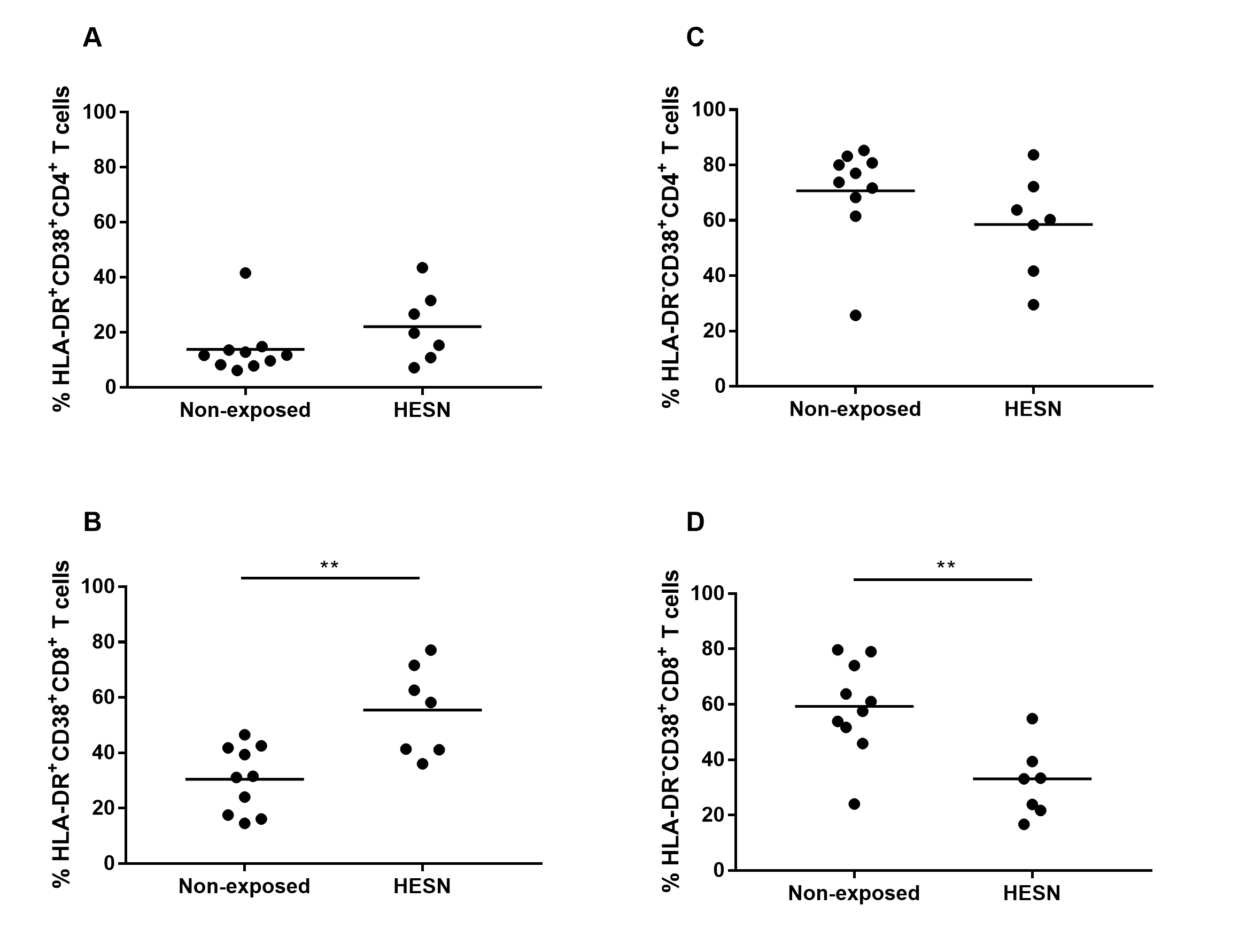

Supplement: S5 Fig — Percentage of HLA-DR+CD38+ in CD4+ (A) and CD8+ (B) T cells. Percentage of HLA-DR+CD38- in CD4+ (C) and CD8+ (D) T cells. Comparison between groups were made by two-tailed, Mann-Whitney test, (*) p≤ 0.05; (**) p≤ 0.01. (TIF) [file pone.0222878.s005.tif]
