# Supplementary material for: Vitamin D treatment of peripheral blood mononuclear cells modulated immune activation and reduced susceptibility to HIV-1 infection of CD4+ T lymphocytes
Source: PLoS One. 2019 Sep 24;14(9):e0222878. doi: 10.1371/journal.pone.0222878 (PMC6759150; doi:10.1371/journal.pone.0222878)
Supplement: S1 Table — (DOCX) [file pone.0222878.s006.docx]

**S1 Table.** Primers sequences for genes evaluated after calcitriol treatment.

| Gene | Primers |
| --- | --- |
| PGK-1 | Fw: 5’-GTTGACCGAATCACCGACC-3’  Rv: 5’-TCGACTCTCATAACGACCCGC-3’ |
| B-ACT | Fw: 5’-CTTTGCCGATCCGCCGC-3’  Rv: 5’-ATCACGCCCTGGTGCCTGG-3’ |
| CYP24A1 | Fw: 5’-CGCAAATACGACATCCAGGC-3’  Rv: 5’-AATACCACCATCTGAGGCGT-3’ |
| VDR | Fw: 5’-TGCTATGACCTGTGAAGGCTG-3’  Rv: 5’-AGTGGCGTCGGTTGTCCTT-3’ |
| IKBα | Fw: 5’-ACACCTTGCCTGTGAGCAG-3’  Rv: 5’-CCATTGTAGTTGGTAGCCTTC-3’ |
| α-DEF | Fw-5’-GCAAGAGCTGATGAGGTTGC-3’  Rv-5’-GTTCCATAGCGACGTTCTCC-3’ |
| HAD4 | Fw: 5’-GCTCTTCAGGTTTCAGGCTCA-3’  Rv: 5’-TCACACCACCAATGAGGCAG-3’ |
| ELAFINA | Fw: 5’ AAACACCTTCCTGACACCATGA 3’  Rv: 5’ TTAACAGGAACTCCCGTGACAG 3’ |
| APOBEC3G | Fw: 5’-CCGTCTGGCTGTGCTACGAA-3’  Rv: 5’-GCTTCCTCCACTTGCTGAACCA-3’ |
| EDN | Fw:5’-GCGGAGACTGGGAAACATGG-3’  Rv:5’-ATTGGTGCATTGCTGGGAGG-3’ |
| ANG | Fw: 5’- CCTGGGCGTTTTGTTGTTGG -3’  Rv: 5’- GGCATCATAGTGCTGGGTCA -3’ |
| SLPI | FW 5’-GATGTTGTCCTGACACTTGTGG-3’  RV 5’-CTTTCACAGGGGAAACGCAGG-3’ |
| RNASE4 | Fw: 5’-GTTGTCATTGCCTGTGAGGGT-3’  Rv: 5’-AGAGCCTGGGACAGCTCAAA-3’ |
| CAMP | Fw: 5’-GGATGCTAACCTCTACCGC-3’  Rv: 5’-AGGGTCACTGTCCCCATACA-3’ |
