# Supplementary material for: Vitamin D treatment of peripheral blood mononuclear cells modulated immune activation and reduced susceptibility to HIV-1 infection of CD4+ T lymphocytes
Source: PLoS One. 2019 Sep 24;14(9):e0222878. doi: 10.1371/journal.pone.0222878 (PMC6759150; doi:10.1371/journal.pone.0222878)
Supplement: S2 Table — (DOCX) [file pone.0222878.s007.docx]

**S2 Table.** Presence of VDREs at the evaluated genes.

| **Gene** | **VDRE presence** |
| --- | --- |
| CD38 | 3 VDRE in promoter region |
| HLA-DR | 4 VDRE in promoter region |
| Granzyme B | 1 VDRE in promoter region |
| IL-2 | 1 VDRE posterior to TSS* |
| APOBEC3G | 5 VDRE in promoter region |
| CAMP | 1 VDRE in promoter region |
| CXCR4 | 1. VDRE posterior to TSS* |

***** TSS – Transcription Start Site
